# Experimental estimation of snare detectability for robust threat monitoring

**DOI:** 10.1002/ece3.3655

**Published:** 2018-01-10

**Authors:** Hannah J. O'Kelly, J. Marcus Rowcliffe, Sarah Durant, E. J. Milner‐Gulland

**Affiliations:** ^1^ Wildlife Conservation Society Cambodia Program Phnom Penh Cambodia; ^2^ Department of Life Sciences Imperial College London London UK; ^3^ Institute of Zoology of the Zoological Society of London London UK; ^4^ Department of Zoology University of Oxford Oxford UK

**Keywords:** detectability, eastern Cambodia, field experiment, snares

## Abstract

Hunting with wire snares is rife within many tropical forest systems, and constitutes one of the severest threats to a wide range of vertebrate taxa. As for all threats, reliable monitoring of snaring levels is critical for assessing the relative effectiveness of management interventions. However, snares pose a particular challenge in terms of tracking spatial or temporal trends in their prevalence because they are extremely difficult to detect, and are typically spread across large, inaccessible areas. As with cryptic animal targets, any approach used to monitor snaring levels must address the issue of imperfect detection, but no standard method exists to do so. We carried out a field experiment in Keo Seima Wildlife Reserve in eastern Cambodia with the following objectives: (1) To estimate the detection probably of wire snares within a tropical forest context, and to investigate how detectability might be affected by habitat type, snare type, or observer. (2) To trial two sets of sampling protocols feasible to implement in a range of challenging field conditions. (3) To conduct a preliminary assessment of two potential analytical approaches to dealing with the resulting snare encounter data. We found that although different observers had no discernible effect on detection probability, detectability did vary between habitat type and snare type. We contend that simple repeated counts carried out at multiple sites and analyzed using binomial mixture models could represent a practical yet robust solution to the problem of monitoring snaring levels both inside and outside of protected areas. This experiment represents an important first step in developing improved methods of threat monitoring, and such methods are greatly needed in southeast Asia, as well as in as many other regions.

## 1 INTRODUCTION

The use of snares is one of the simplest but most effective hunting techniques practised in the tropics (Fa & Brown, 2009). Despite the threat posed to mammals by this form of hunting (Corlett, 2007; Harrison et al., 2016), reliable assessments of snaring prevalence within protected areas are practically nonexistent. One of the primary reasons for this is that rigorous methods for estimating the extent of snaring have not yet been developed. Studies have addressed snaring levels within some African protected areas (e.g., Becker et al., 2013; Wato, Wahungu, & Okello, 2006), but these studies are susceptible to bias arising from low effective sampling effort, nonrandom sampling, and failure to account for imperfect detection within sample plots. This is because snares share many of the characteristics of the species they target; they are habitat specific, extremely difficult to detect, and occur in remote, inaccessible areas. And, just as for rare species in the tropics, traditional methods to obtain unbiased population estimates are extremely difficult to implement in these conditions.

Although the practice of snaring is pervasive within protected areas throughout the tropics, snares are well concealed by the hunters who set them, and because of the large size of many of these areas, they occur at a relatively low density overall. As a result, attempts to estimate snare abundance are fraught with both statistical and practical difficulties, and the detection probability for snares is likely to be low. Simple plot sampling methods could be used for snares (as are commonly used for plant populations), with plots chosen according to some probability‐based sampling design (Williams, Nichols, & Conroy, 2002). However, the logistical burden of establishing many small plots across the entirety of a typical protected area renders this unfeasible in most situations. Furthermore, there would be a substantial risk that sampling within many small random or systematically placed plots would result in few or no snares being observed. This same limitation applies to attempts to use fixed transect surveys for snares.

One potentially practical solution is an approach involving larger sample “plots” in which surveys teams are allowed to search purposefully. Teams can then use landscape features and other cues, such as hunter trails, to focus search effort and optimize search efficiency. This approach seems promising, but as with any new method, field testing is essential. One obvious issue to address is the fact that not all snares that are present will be observed by survey teams, and any proposed survey method must provide a means of accounting for imperfect detection.

The problem of imperfect detection within ecological surveys is widely acknowledged (Kellner & Swihart, 2014; Yoccoz, Nichols, & Boulinier, 2001) and ignoring this issue can result in biased estimates that may lead to misinformed management decisions (Guillera‐Arroita, Lahoz‐Monfort, MacKenzie, Wintle, & McCarthy, 2014; Kéry & Schmidt, 2008). A range of methods have been developed to account for imperfect detection, from classic distance sampling methods (Buckland et al., 2001, 2004) and capture–recapture methods (Otis, Burnham, White, & Anderson, 1978; Pollock, 1982) to the more recent occupancy methods (MacKenzie et al., 2002, 2006; Tyre et al., 2003). A common feature of these methods is that they generally require data to be collected such in a way that allows the detection process to be modeled explicitly, and separately, from the ecological process of interest, be that occurrence, abundance or species richness (Kéry & Royle, 2016; Royle & Dorazio, 2008). Such hierarchical models are commonly used within studies of plants and animals, but to our knowledge, they have never been applied to surveys for snares.

The primary objective of this study was to determine the detection probability for snares in an experimental field context in which the true abundance of snares was known. It was anticipated that this would yield valuable baseline data on the potential detection probability of snares in a tropical forest setting, and also facilitate an assessment of how various factors might affect this detection probability. This type of information is critical for the future development of more large‐scale systematic snare surveys, which could function as part of a threat monitoring program aimed at assessing protected area management effectiveness.

Within a controlled environment, it was possible to observe directly whether and to what extent the detection probability of snares varied between snare types, habitat types, and observers. We hypothesized that detectability would be lower in more closed habitats (evergreen forest) than in open habitats (mixed forest) and that groups of snares (snare lines) would have a higher detection probability than single snares. As the teams participating in this experiment had similar levels of skill, and were all highly motivated, we expected that detection probability would be relatively consistent across teams.

A secondary objective of this study was to compare the feasibility of implementing two candidate sets of sampling protocols and associated hierarchical modeling techniques. The sampling protocols of interest were a simple repeated count approach (e.g., Kéry, Royle, & Schmid, 2005) and a double‐observer approach (e.g., Alldredge et al., 2008; Nichols et al., 2000). The associated modeling approaches being tested were, respectively, a binomial mixture model (Royle, 2004a,2004b) and a multinomial mixture model (Kéry & Royle, 2016). As opposed to simple counts, the double‐observer approach requires that snare events can be identified individually, in order to determine which were detected by only one observer and which were detected by both. This entails additional design considerations and has implications for any monitoring programme based on either of these approaches, as managers will place a high priority on logistical feasibility and cost‐effectiveness (Jones, 2011). In addition, there are ethical concerns implicit in not removing snares allow for their detection by subsequent observers.

As this field experiment relied upon a small sample size, the emphasis in this second objective was on investigating if and how practical field sampling protocols could be developed which allowed for individual identification of snares, rather than a comparison of the estimates produced from the associated modeling process.

## 2 METHODS

### 2.1 Modeling framework

Binomial mixture (or N‐mixture) models (Royle, 2004a,2004b) and multinomial mixture models (Kéry & Royle, 2016) were developed to analyze data collected at multiple different locations, or “sites,” which is frequently the case in ecological studies. Both types of model use replication, through the sampling of multiple sites and additional repeated measures *within* sites, to separate out the detection process from underlying abundance. However, with multinomial mixture models, it is necessary to be able to identify individuals across repeated measures within sites, whereas with binomial mixture models, there is no such requirement (Kéry & Royle, 2010, 2016). Multinomial mixture models would be expected to provide more precise estimates of abundance due to the extra information incorporated in the modeling process (Kéry & Royle, 2010, 2016), but individual identification of snares may not be practically feasible in some circumstances and where it is, may increase sampling costs in terms of time or effort. Both models assume population closure during the survey and that sampling is random. The N‐mixture model includes the additional assumption that detections are independent and that individuals (or snare events in this context) have same detection probability within a site.

### 2.2 Experimental strategy

To directly observe the detection probability of snares, it was necessary to create an artificial closed “population” of snares and then deploy field teams to search for these snares. The snares were distributed across multiple large plots in a manner that was as realistic as possible. Multiple teams searched each site, independently of one another. Teams did not follow fixed routes but attempted to maximize the probability of encountering a snare. This meant that they were able to follow apparent hunter trails and focus attention on features known to be favoured by hunters for setting snares, such as streams and hillsides. Each team corresponded to an “observer” and the snare events encountered by teams comprised the count data. The experiment was designed in such a way that all of the following could be clearly determined from data: (1) The positions of all available snare events; (2) the search routes followed and snare events found by each team; and (3) the instances where multiple teams encountered the same snare event.

### 2.3 Sampling protocols

Two sets of sampling protocols were being tested during this experiment, both of which are standard practise within ecological surveys. The first of these is a simple repeated count approach, where multiple observations/visits are carried out at spatially replicated sites (e.g., Kéry et al., 2005). The second is a double‐observer approach (e.g., Alldredge et al., 2008; Nichols et al., 2000) where counts are made by two observers simultaneously, again at spatially replicated sites, and counts are compared to identify individuals encountered by both observers. Notably, this independent double‐observer approach is exactly analogous to a capture–recapture model with two sampling occasions, with each observer being equivalent to an occasion.

### 2.4 Sampling locations

This experiment was conducted over a week‐long period in October 2011, in the core area of the Keo Seima Wildlife Sanctuary (KSWS), a 292,690 ha protected area in eastern Cambodia. Two sampling locations were selected, one in mixed deciduous forest and another in evergreen forest, thereby representing the dominant habitat types within the reserve. A total of 22 plots or sites were delineated, each measuring 1 km by 1 km. Twelve were located in mixed forest and ten in evergreen. The sites were identified by marking them out on a topographical map and subsequently by inputting the relevant UTM coordinates into handheld Garmin GPS units.

### 2.5 Survey teams

A total of seven teams participated in the experiment, each consisting of one experienced team leader from the permanent biological monitoring team working within the KSWS and two local assistants from villages within and around the core area. When recruiting local assistants, team leaders attempted to seek out individuals who had experience of hunting, and in particular hunting with wire snares, within the core area. This was in order to maximize detectability as these individuals have knowledge of where snares are likely to be placed and how to find them.

### 2.6 Field implementation

**Figure 1:**
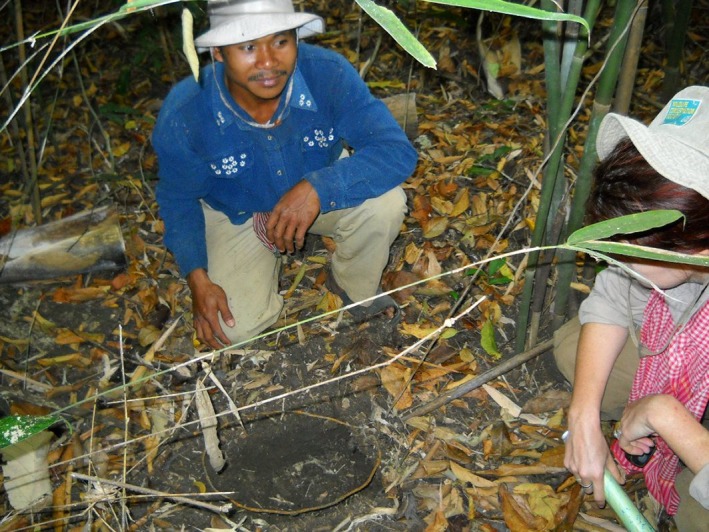
Single snare (with covering of leaf litter removed)

Snares are typically constructed using a looped brake cable or similar type of wire which is buried under leaf litter or suspended just above the ground (Figure 1). The loop is attached via another length of wire to an anchor pole, usually a strong flexible sapling which is firmly fixed in the ground. A simple trigger mechanism is sometimes incorporated, built into the ground and activated by an animal stepping through on it. In this experiment, it was necessary to ensure that no animals were inadvertently captured or injured and so it was not possible to use real snares. Instead, plastic string was used rather than wire, and this was loosely attached to an anchor pole, with no trigger mechanism. These replica snares were divided into two types, reflecting the types of snare commonly used for hunting in the KSWS. Single large snares were set individually within dense undergrowth while groups of smaller snares were set at intervals along a low brushwood drift fence, designed to guide prey into the snares. The intention was that replica snares looked similar enough to the genuine article to mimic the detection process for search teams, but to present no danger to wildlife in the area.

Sampling took place over a two‐day period at the mixed forest location and over a three‐day period at the evergreen forest location, due to the more difficult terrain which characterized the latter. This slight difference in timing was judged not to effect detectability in this environment. On the first day at each location, each team was allocated a number of sites together with the equipment to construct the dummy snares. The teams were instructed to set a randomly assigned number of snares, between 1 and 20 (as a best‐guess approximation of what actual snare densities might be). Each team decided on the distribution of snares across their sites, and they were encouraged to choose the best position and the most appropriate type of snare (i.e., single snare or snare line) according to their own previous experience of encountering (or using) snares within the KSWS. Both single snares and snare lines were considered equivalent “snare events” for this experiment. Although they did not necessarily have to set snares at all sites, all teams chose to do so. Each team was supplied with a detailed topographical map and used Garmin GPS units to navigate within their assigned sites, to record the locations of the snares they had set, and to track their exact routes. Finally, all team members were also instructed not to disclose or discuss the locations of their set snares with other teams.

On the subsequent days (day two for the mixed forest sites and days two and three for the evergreen forest sites), teams were again assigned a number of sites, chosen randomly with the exception that no team would search a site that it had set snares in on day one. Each site was surveyed by two separate teams independently (both teams on the same day in the mixed forest sites and on consecutive days in the evergreen sites), and each team was required to aim for approximately two kilometers of walk effort within each site, following a route of their own choosing. They used the topographical maps and their knowledge of where snares are likely to be set to search each site to the best of their ability. Team leaders were primarily concerned with navigation and data recording while local assistants searched for snares. Teams recorded the UTM coordinates of all encounters and although they recorded whether it was a single snare or a snare line, both counted as snare events (i.e., multiple snares in a line were counted as one event). Their precise route was recorded by the GPS tracklog function. Teams took care not to disturb vegetation or to leave traces which might be used by subsequent teams to locate the snares, and to leave all snares that they found intact.

### 2.7 Analysis

The GPS data, relating to all snares set and snare events found by teams, together with the routes taken by teams, were downloaded and examined in ArcGIS software. First, the number of snare events actually detected was compared to the number of snare events available for detection, in order to determine the “true” detection probability in this experimental context. Snare events which were detected by both survey teams were also identified at this stage. The data were subdivided by snare type (single or snare line), team, and by habitat to explore potential differences in detection probability. For this part of the analysis, individual snare events were aggregated across all sites (by type, habitat and team) so that no distinction was made between sites. In addition, GPS tracklog routes were also examined visually within each site to compare how each of the two survey teams searched.

Second, aggregated counts of snare events within each individual site were tabulated for the first and second survey teams. These data were analyzed in the “unmarked” package (Fiske & Chandler, 2011) in R version 2.14.0 (R Core Team, 2012). Due to the limited quantity of data available, no distinction was made between habitat, teams or snare type for this analysis, although this would be possible with a larger dataset. Two separate fitting functions “pcount” and “multinomPois” were used, representing the binomial mixture model and multinomial mixture model, respectively. The detection process was modeled as binomial in the first approach, whereas a multinomial distribution was used for the detection process in the second approach. For both approaches, a latent Poisson distribution was assumed for abundance at each site, although alternative distributions can be specified (Kéry & Royle, 2016).

Binomial mixture models do not require the unique identification of individual snare events across visits (although within visits individual events must be identifiable to avoid double‐counting) so, for the purposes of this model, where two teams both encountered a given snare event, the detections were assigned to both visits but were not associated with each other. With the multinomial model, each visit was treated as an independent count but one where snare events could be uniquely identified and matched if counted by another team.

## 3 RESULTS

Over all 22 sites, a total of 115 snare events (including both single snares and snare lines) were set, 35 of which were detected by at least one team, and 11 of which were detected by both teams (Table 1). Slightly fewer than 40% of available snares were detected in evergreen forest sites, while just over 20% of snares were detected in mixed forest sites. The proportion of available snare lines detected was 10% greater than single snares, and this trend was observed in both mixed forest sites and evergreen forest sites (Table 1). The proportion of snares detected in the first and second visits was similar, although it was slightly higher for the second visit, in both habitat types (Table 1).

**Table 1:** Number and type of snares set (i.e., available for detection) in evergreen (EVG) and mixed (MF) forest, number of snares found by all teams over first and second visits, and associated detection probabilities for each

Snare/habitat type	No. set	Found Pass 1	Found Pass 2	Found at least once	Found both times	Detection Pass 1	Detection Pass 2	Overall Detection
MF snare line	20	3	4	5	2	0.15	0.2	0.18
MF single snare	38	4	6	8	2	0.11	0.16	0.14
MF ALL	58	7	10	13	4	0.12	0.17	0.15
EVG snare line	22	5	8	10	3	0.23	0.36	0.3
EVG single snare	35	9	7	12	4	0.26	0.2	0.23
EVG ALL	57	14	15	22	7	0.25	0.26	0.26
ALL	115	21	25	35	11	0.18	0.22	0.2

Due to the high number of teams and relatively small number of sites, there is little information to definitively identify differences in detection probability between teams. This is further complicated by the nonequal allocation of sites between teams (for logistical reasons), as well as the variability in the number of snares available for detection within these sites. While the limited data show some variation in the proportion of snares detected by different teams, ranging from 11% to 30% (Table 2), this difference is not statistically significant (χ^2^ = 5.6, *df* = 6, *p* = .47).

**Table 2:** Variation between teams, aggregated across both habitat types and snare types

Team	Total available snares	Total snares found	Detection
A	42	9	0.21
B	40	5	0.13
C	42	10	0.24
D	18	2	0.11
E	23	7	0.3
F	36	9	0.25
G	29	4	0.14

**Figure 2:**
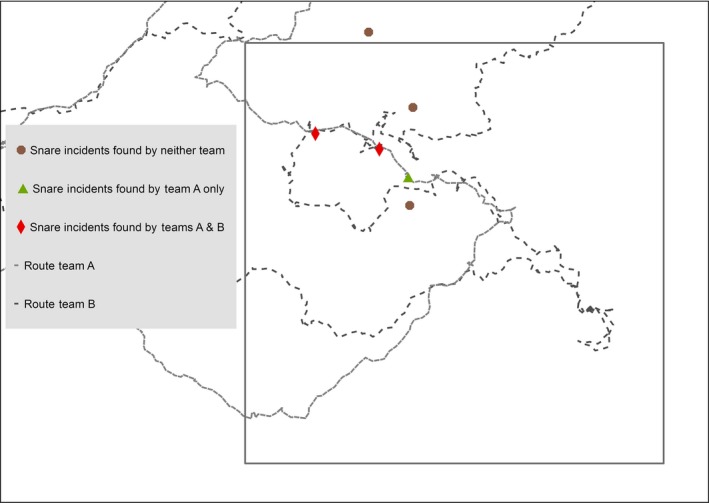
Tracklogs showing survey routes taken by independent teams within one mixed forest site. The locations of snares events available for detection, and snares detected by one or both teams are also shown

The visual inspection of GPS tracklogs revealed that teams did overlap in their survey routes but not to any major extent (see Figure 2 for an example). The teams surveyed both on and off existing trails and survey routes tended to coincide on trails. The degree of overlap observed between teams also appeared to be greater within evergreen forest sites when compared to mixed forest sites.

An additional point worth noting is that eight genuine single snares (i.e., snares which had been placed independently by hunters not involved in this experiment), and one real snare line were detected by teams during the course of this experiment. The snare line was encountered by both teams that surveyed the site, while the eight single snares were each encountered by only one team. All of these snares were disabled by removing the wires, although the drift fence structure remained in place.

The results of the two hierarchical models are presented in Table 3. The binomial mixture models results, which are derived from the simple repeated counts, appear closer to “true” detection probability and abundance, when compared to the multinomial mixture model results, which are based on the double‐observer method. Both approaches overestimated detectability, and underestimated mean abundance per site, the multinomial mixture model more so than the binomial. The standard errors associated with the estimates were higher, however, for the binomial mixture model than for the multinomial mixture model.

**Table 3:** Model estimates from two approaches compared to” true values”

Model	Binomial mixture	Multinomial mixture	True value
Abundance estimate	3.39	2	5.2
*SE*	1.72	0.43	
Detection probability	.28	.48	.2
*SE*	0.14	0.09	

True values are mean abundance per site (dividing total number of snares set by total number of sites).

## 4 DISCUSSION

An overall detection probability of 0.2 in this experimental context supports the supposition that detection rates for snares are low. In most ecological surveys, only a small proportion of the total area will be sampled, and in the case of snares, realistic search effort may allow only a small proportion of the total number of snares present to be detected. Had the teams not been allowed to search purposefully (e.g., if they searched along random transects), the detection probability would probably have been far lower.

As predicted, snare lines were more conspicuous than single snares and this is reflected in their higher detection probability in both habitat types, and particularly in dense evergreen forest where single snares are especially difficult to pick out. Surprisingly, however, overall detectability of snares was higher in evergreen forest sites than in mixed forest sites, which is contrary to expectations. A possible explanation is that within evergreen sites, the difficult terrain means that teams are to a greater extent constrained in their choice of routes. While in more open mixed forest sites, teams can traverse the site freely, teams both setting and searching for snares in the evergreen forest may be forced to follow similar paths—as they are the only ones available. The fact that a greater number of double‐detections occurred in the evergreen forest than the mixed forest sites (seven versus four) may bear this out.

This raises the issue of whether teams may be cueing each other more generally, for example, by tracking one another or noticing greater disturbance around the vicinity of a snare (because teams have stopped at these locations). This does not seem likely in this context; however, as these areas are heavily used by local communities for a wide range of resource collection activities, meaning multiple trails exist and levels of disturbance are high throughout the area. In addition, the examination of tracklog routes did not appear to show an overwhelming degree of overlap between teams. The overlap does appear to be greater within evergreen sites, but this again may be due to the more restricted movement in this habitat. The proportion of snares detected on the second visit is slightly higher than on the first visit, to sites in both habitat types, and it is not possible to entirely discount the possibility that teams were to some degree tracking one another. However, it does not appear particularly plausible that survey teams would have been able to distinguish between signs or tracks resulting from normal community members' activity and those caused by their fellow survey teams.

It is also pertinent that in any survey conducted to locate “real” snares, search teams will inevitably follow cues they believe to be related to the movement of hunters, that is, trails and camps, and in fact, this is the most efficient method to detect snares. Thus, in reality, this is a technique search teams *should* be employing, even in this experiment. The vast majority of ecological surveys are concerned with nonhuman species but in studies like this, where “signs” of illegal human activity are the target, it may be practical to capitalize on the fact that search teams will naturally tend to navigate in a similar manner to the perpetrators of such activities.

In this experiment, we attempted to mimic the field conditions in which a real snare survey might be undertaken to as great an extent as possible. However, although the teams involved in setting snares for this experiment included individuals with hunting experience, in this scenario, they were not actually intent on catching prey and avoiding capture themselves. Furthermore, snares occurred at moderately high densities and were distributed quite evenly across sites within this experimental scenario. In a real survey context, there is likely to be greater spatial heterogeneity in terms of snare distribution and densities could be either far lower or, indeed, far higher locally. Hunters may systematically avoid areas in which they know other hunters operate or which are regularly patrolled by law enforcement teams. They may also employ different strategies for and intensities of snare placement, depending on prey preference etc.

Typically in a double‐observer approach, both teams (i.e., observers) will search simultaneously while repeated visits are generally carried out consecutively. In this experiment, some sites were searched on the same day and some on successive days. Given that the availability of snares for detection was not expected to change over a two‐day period, both approaches are exactly equivalent in terms of sampling protocols in this context. It was of interest in this experiment to determine whether teams searching in this manner would achieve multiple detections of the same snare event, which would provide more information for parameter estimation, or if simple repeated counts might be a more realistic approach. Both sampling protocols appeared to be feasible in this context, but both also involved teams detecting snares and, rather than removing them, leaving them for detection by additional teams. This raises obvious ethical concerns, and in any real snare survey, some means of destroying the snares while still leaving a cue for subsequent survey teams would need to be developed.

For the modeling process, the multinomial model used for the double‐observer approach incorporates an extra layer of information, that is, the double‐detections, which are discarded for the simple repeated count approach using the binomial model. The former was expected to prove a better estimator given sufficient “recaptures,” but it was of interest to see how the latter performed, as identifiable repeat encounters of the same snare may not occur in other situations. In fact, contrary to predictions, the simple repeated count model appeared to provide more accurate estimates of detectability and mean abundance per site. It is important to note, however, that in the context of this experiment, this modeling exercise was exploratory in nature and results must be interpreted with caution. There was evidence of heterogeneity in detection probabilities which would constitute a violation of the assumptions underlying N‐mixture models (Veech, Ott, & Troy, 2016). The inclusion of covariate data in future surveys could help to address this issue, but larger sample sizes and additional covariate data would be required. For the double‐observer approach, it is possible that the small sample size introduced bias into the estimates, but it is also possible that the overestimate of detectability may be a result of some degree of nonindependence between teams. This could have arisen because teams had very similar ideas with regard to where to both set and search for snares.

## 5 CONCLUSION AND RECOMMENDATIONS

Tracking trends in threats is often a critical component of any monitoring program. Not only does threat monitoring provides a means of assessing the effectiveness of management interventions, but it can provide information in the medium term, perhaps before impacts on biological populations become measurable (Kapos et al., 2008; Salafsky, Margoluis, Redford, & Robinson, 2002). However, threats or threat indicators are not always detected perfectly and, as with biological targets, any method for monitoring threats must address this issue. Failure to do so risks producing biased estimates, which can in turn lead to nonoptimal or even detrimental management decisions (Legg & Nagy, 2006; Nichols & Williams, 2006).

Prior to this study, there was virtually no information available relating to the practice of snaring which could help inform the design of any large‐scale assessment of snaring prevalence. More generally, despite the wide array of methodologies available for population estimation and trend monitoring, many remain untested in tropical forest settings, both with regard to the validity of underlying model assumptions and also in terms of practical feasibility in challenging field conditions. Furthermore, these methods have been developed primarily for use with animal populations and the fact the snares are essentially indirect signs of human activity adds an additional element of complexity to be considered when selecting an appropriate design.

Although this experiment focused on an artificial scenario, it has provided a preliminary estimate of the detection probability of snares in a tropical forest setting and yielded useful insights into what factors might affect snare detectability, information which can be used to guide the design of future surveys. It is clear that methods to reliably estimate snaring levels and monitor changes in these levels must take into account imperfect detection and that method which do so can be implemented in such challenging contexts. However, further research is needed in this area. Surveys with larger sample sizes and which include covariate information are necessary. It would also be particularly useful to test additional sampling protocols that would enable snares to be removed by the first team they are encountered by, such as removal methods, and time‐to‐detection methods.

## CONFLICT OF INTEREST

None declared.

## AUTHOR CONTRIBUTIONS

HJOK conceived of the study, collected and analyzed the data, and authored the manuscript. EJMG, JMR & SD provided substantial input into the study design and data analysis, and revised the manuscript.
